# Lytic or Latent Phase in Human Cytomegalovirus Infection: An Epigenetic Trigger

**DOI:** 10.3390/ijms262311554

**Published:** 2025-11-28

**Authors:** Armando Cevenini, Pasqualino De Antonellis, Laura Letizia Mazzarelli, Laura Sarno, Pietro D’Alessandro, Massimiliano Pellicano, Serena Salomè, Francesco Raimondi, Maurizio Guida, Giuseppe Maria Maruotti, Marco Miceli

**Affiliations:** 1 Department of Molecular Medicine and Medical Biotechnology, University of Naples Federico II, 80131 Naples, Italy; pasqualino.deantonellis@unina.it; 2 CEINGE–Biotecnologie Avanzate Franco Salvatore, 80145 Naples, Italy; 3 Department of Public Health, University of Naples Federico II, 80131 Naples, Italy; lauramazzarelli@gmail.com (L.L.M.); pietro.dalessandro9676@gmail.com (P.D.); giuseppemaria.maruotti@unina.it (G.M.M.); 4 Department of Neuroscience, Reproductive Sciences and Dentistry, University of Naples Federico II, 80131 Naples, Italy; laura.sarno@unina.it (L.S.); pellican@unina.it (M.P.); mauguida@unina.it (M.G.); 5 Division of Neonatology, Department of Translational Medical Sciences, University of Naples Federico II, 80131 Naples, Italy; serena.salome@unina.it (S.S.); francesco.raimondi@unina.it (F.R.); 6 Regional Reference Center (CER) for Infectious Diseases in Obstetrics and Gynecology in Campania, 80131 Naples, Italy

**Keywords:** human cytomegalovirus (HCMV), epigenetics, latent phase, lytic phase, histones

## Abstract

Human cytomegalovirus (HCMV) is a herpesvirus (family) belonging to the beta herpesvirus subfamily that causes significant morbidity both in immunocompromised hosts (horizontal transmission) and during vertical transmission from mother to child. HCMV has the ability to establish a permanent latent infection with its host (even for decades), in which the DNA remains as a silent nuclear episome (latent phase) until reactivation after the appropriate conditions have occurred (lytic phase). The transition between the two phases (latent/lytic) is largely determined by the type of infected cell and the health status of the host, which ultimately corresponds to the epigenetic state of the infected cells. Lytic infection of the virus normally occurs in epithelial cells, endothelial cells, fibroblasts or macrophages, whereas the latent phase occurs when undifferentiated cells of the myeloid lineage, such as CD34+ hematopoietic progenitor cells, are infected. Epigenetic regulation of the viral genome begins with the formation of chromatin in the viral DNA just 30 min after infection and then evolves towards the latent or lytic phase. DNA viruses, including members of the herpesvirus family, are currently the subject of intense study regarding the role that epigenetics plays in controlling the viral life cycle, focusing primarily on the role of post-translational modifications (PTMs) of histones, as well as DNA methylation. Within the viral genome, nucleosomes are organized for the spatial/temporal expression of appropriate genes due to epigenetic modifications. Therefore, during the infection cycle, DNA chromatinization and chromatin modifications influence the expression of genes in the HCMV genome. This process is mediated by (i) enzymes called “writers”, which catalyze PTMs by adding chemical groups to proteins (acetylation, methylation, etc.); (ii) recruitment of specific transcription factors called “readers”, that bind to modified amino acid residues of proteins and act as interpreters of the PTM code; and (iii) “erasers”, enzymes that remove these modifications (e.g., HDACs). Indeed, recent advances in understanding the chromatin-based mechanisms of viral infections offer some promising strategies for therapeutic intervention that could be particularly useful in immunosuppressed recipients of transplants to avoid allograft rejection and infection by other opportunistic pathogens. In this review, we comprehensively examine the epigenetic regulation of the HCMV genome across distinct phases of viral infection, with particular attention to recent studies that significantly enriched the current knowledge about molecular mechanisms and future therapeutic perspectives.

## 1. Introduction

Epigenetic regulation plays a central role in controlling the life cycle of herpesviruses, including human cytomegalovirus (HCMV). Like cellular chromatin, viral genomes are subjected to DNA methylation, histone modification, and nucleosome organization, which collectively determine transcriptional activity and the balance between latency and lytic replication [1]. Upon nuclear entry, the naked HCMV genome rapidly associates with host histones, forming chromatin whose transition to a euchromatic or heterochromatic state determines if the infection will proceed towards the lytic or the latent phase, respectively [2]. The transition between these two phases is mainly due to the type of infected cell and the health status of the host, which in turn define the epigenetic state of the infected cells. Indeed, permissive cells (e.g., epithelial cells, endothelial cells, fibroblasts, and macrophages) support lytic replication through euchromatin formation, whereas non-permissive cells, such as CD34^+^ progenitors and CD14^+^ monocytes, favor the establishment of a latent phase characterized by heterochromatic viral DNA [3,4].

During lytic infection, viral immediate early (IE) genes drive the transcriptional cascade necessary for viral replication, antagonizing host repressors such as histone deacetylases (HDACs) and promoting histone acetylation and chromatin relaxation [5].

In contrast, during latency, the viral genome becomes largely heterochromatinized, inaccessible to transcription factors thanks to the recruitment of corepressor complexes composed of enzymes that catalyze repressive histone modifications, compact chromatin, and prevent nucleosome remodeling. Thus, the viral chromatin is enriched in repressive histone marks and is maintained in a transcriptionally silent state by host factors such as Polycomb repressor complexes. Viral factors inhibit the chromatin demethylation, thereby stabilizing its repressive state, and recruit repressive complexes to viral gene promoters to reinforce silencing [6].

Reactivation from latency involves chromatin remodeling, characterized by increased histone acetylation, loss of repressive methylation, and activation of the major immediate early (MIE) promoter. Viral and host transcription factors coordinate this transition, leading to euchromatin formation and productive replication [7].

Unfortunately, to date, our knowledge of the exact basic mechanisms for maintaining viral latency and for the reactivation from latency remains incomplete and fragmented despite numerous studies. However, in recent years some significant progress has been made and recent advances in understanding the chromatin-based mechanisms of viral infections are offering some promising strategies for therapeutic intervention. These approaches are mostly aimed at avoiding lytic phase reactivation in the case of immunocompromised hosts, such as immunosuppressed recipients of transplants, where HCMV infection increases the risk of acute and chronic allograft rejection and infection by other opportunistic pathogens [8,9,10,11,12].

In this review we aim to summarize the current knowledge about the links between epigenetics and molecular regulation of the HCMV lifecycle, with a particular focus on the mechanisms driving the transition between the lytic and latent phases of the infection. Thus, in the first sections we provide a summarized but comprehensive overview of (i) epigenetics; (ii) biological events that follow one another during a viral infection, with a particular focus on herpesviruses; (iii) general characteristics of HCMV, including some clinically relevant aspects; and (iv) the HCMV lifecycle, including gene regulation of the lytic phase and chromatinization of the viral genome. Then in the subsequent sections we focus on the intricate interplay between viral and host epigenetic machinery in regulating the HCMV latent–lytic switch; we hereby describe the knowledge about the mechanisms that (i) overcome the repression of the HCMV genome, (ii) favor the reversion from the latent to the lytic state, (iii) reactivate viral gene transcription, and (iv) lead to lytic viral replication. Finally, in the last section of this review, we highlight how recent advances in the understanding of the epigenetic determinants of the latent–lytic state are leading to potential therapeutic strategies to prevent or limit the lytic phase reactivation, especially in immunocompromised patients.

## 2. An Overview of Epigenetics

Viruses, including herpesviruses, are currently being studied for the role epigenetics plays in controlling the various stages of their life cycle, focusing primarily on histone modifications, nucleosome localization, and DNA methylation.

Despite substantial diversity in genome composition (RNA vs. DNA, single- vs. double-stranded), viruses employ conserved epigenetic strategies to regulate transcription. Upon nuclear entry, many viral genomes are chromatinized into nucleosomal structures that undergo canonical histone modifications (e.g., acetylation, methylation, phosphorylation, sumoylation). These modifications modulate chromatin accessibility and enable precise spatial and temporal control of viral gene expression, closely paralleling mechanisms in eukaryotic chromatin [2].

The term “epigenetics” refers to the modulation of gene expression in heritability, development, cell fate, and cellular programming/reprogramming, without changes to DNA sequence [13], according to highly complex and structured molecular codes. Aberrations in epigenetic effectors that mediate covalent modifications in gene regulatory regions and in the histone proteins around which viral DNA is wrapped can lead to adjustments in the epigenetic configuration as different phases of the viral life cycle unfold.

Since the biologically functional state of DNA in eukaryotes, and consequently in viruses (obligate intracellular parasites), is represented by chromatin, in which DNA is wrapped around a histone core. Epigenetic regulation operates primarily through DNA methylation and demethylation, as well as post-translational modifications of histones H2A, H2B, H3 and H4 (including acetylation, methylation, phosphorylation, sumoylation, etc.) [2].

Epigenetic regulation of DNA occurs through the covalent addition of a methyl group to the cytosine of the CpG sequence [3,14] by the eukaryotic DNA methyltransferases: Dnmt1, Dnmt2, Dnmt3a, and Dnmt3b. Dnmt1 is responsible for maintaining DNA methylation during replication, preferentially acting on hemimethylated DNA. Dnmt3a and Dnmt3b are primarily responsible for de novo DNA methylation, i.e., establishing DNA methylation patterns during development and in response to environmental stimuli [4,15], while the biological function of Dnmt2 is still under study [5,16]. DNA methylation is generally believed to be associated with the repression of gene expression [3,14].

Histone proteins epigenetically influence viral chromatin primarily through post-translational modifications of their amino acid residues but also through the insertion of histone protein variants into the histone core. Histones H2A and H3 exist in multiple forms that differ in their primary amino acid sequence at a limited number of protein sites [6,17]. These modifications generally consist of the substitution of serine with valine or alanine in regions other than the amino-terminal region.

There are four well-known variants of H3 (H3.1, H3.2, H3.3, and CENP-A) and three of H2A (H2A.Z, H2A.X, and macroH2A). The H3 variants H3.1 and H3.2 are primarily associated with newly synthesized chromatin in higher eukaryotes. H3.3 is associated with chromatin undergoing active transcription. CENP-A (CENH3) is restricted exclusively to the centromeric regions [7,18]. The interaction of DNA with the histone core in chromatin formation is highly dynamic. Indeed, during all processes in which DNA sequence information is read (replication, transcription, DNA repair, etc.), specific proteins intervene to interrupt and then restore the DNA/histone core interaction. Nucleosome formation typically involves DNA, the four canonical histone proteins (H2A, H2B, H3, and H4) that form the histone core or their variants (H3.1-2-3, CENP-A, etc.), specific chaperones, and various proteins (involved in DNA replication or transcription). For example, H3.1 interacts with CAF-1 and ASF-1 through interaction with PCNA at the replication fork [19], while H3.3 interacts with the histone cell cycle regulator A (HIRA) during transcription, and CENP-A interacts with the Holliday junction recognition protein (HJURP), which recognizes the centromeric region [19].

The H2A.X variant is primarily found active during DNA repair, while macroH2A appears to be associated with transcriptionally repressed regions (constitutive heterochromatin). Finally, H2A.Z appears to be enriched at the flanks of nucleosome-free regions [20].

Numerous studies have shown that post-translational modifications of histones (acetylation, methylation, phosphorylation, ADP-ribosylation, ubiquitination, etc.) play an important role in epigenetic regulation. Acetylation of H3-lysines (9 and 14) and H4-lysines (5, 8, 12, 16, and 20) is mainly associated with transcriptionally active chromatin in a dynamic acetylation/deacetylation process during RNA polymerase II activity [21]. Several well-characterized histone acetyltransferases (HATs), including p300, CBP (CREB-binding protein), and GCN5 (General Control Non-depressible 5), catalyze histone acetylation [22]. Similarly, at least 18 HDACs (Histone deacetylases) are known, divided into four classes based on their sequence and function: Class I (HDAC1, 2, 3 and 8) is constituted by orthologs of the yeast enzyme Rpd3 (Reduced Potassium Dependency 3); Class II (HDAC4, 5, 6, 7, 9 and 10) contains orthologs of the yeast enzyme Hda1 (histone deacetylase 1); Class III (the Sirtuin family 1-7) contains orthologs of the yeast enzyme Sir2 (silent information regulator 2) [23,24]; Class IV encompasses only one known member, namely HDAC11, which does not have an ortholog in yeast [25] (Table 1).

H3 and H4 methylation is the other major form of histone PTM, in which a particular lysine can be mono-, di-, or tri-methylated, depending on the cell type, differentiation stage, and location within the genome, significantly increasing its complexity. For example, trimethylation of H3K4, H3K36, and H3K79 is predominantly associated with transcriptional activation, whereas trimethylation of H3K9, H3K27, and H4K20 is commonly associated with transcriptional repression [26]. Furthermore, the monomethylated form of many of these modifications can have a regulatory effect opposite to the trimethylated form [27]. Numerous histone methylases have been identified, and in general, each form of histone methylation is mediated by its own enzyme [26]. Likewise, enzymes capable of demethylating each of the methylated forms of histones (such as the MBD (Methyl-CpG-Binding Domain) family) have been characterized [26].

## 3. Biological Events That Occur During an Infection

The biological processes, including epigenetic ones, that occur during infection by a DNA virus from the herpesvirus family follow two distinct pathways. The first pathway triggers a productive infection with the generation and assembly of viral progeny (lytic phase), while in the second pathway, or latent phase, the virus enters the cell in a “frozen” form in which the viral genome forms a stable, transcriptionally repressed episome.

But let us proceed in order: the initial phases of a typical dsDNA virus infection include binding to a receptor on the external cell surface, internalization of the virion with subsequent uncoating, and finally transport to the cell nucleus. Once it reaches the nucleus, the viral genome undergoes the activation of certain genes necessary for the initiation of infection (immediate early genes) [2].

The initiation of viral transcription depends on the specific virus type. Some viruses, such as SV40, are present in virions as chromatin and are able to carry transgenerational epigenetic information [28]. Other viruses, such as herpesviruses, enter the cell as “naked” DNA, which is then organized into chromatin once released into the nucleus [29].

After transcription begins, both in viral and cellular genomes, regulatory processes occur that modulate gene expression, i.e., activate or repress specific genes. These regulatory events are crucial in determining cell fate, the response to external stimuli, and the viral replication cycle. The order of these events during infection can vary depending on the specific virus type. For example, some viruses, including members of the herpesvirus family, can exist within the cell in multiple stages of their life cycle, including a latent state in which transcription is minimal and the viral chromatin remains long-term stable in the form of an episome. In other cases, viruses integrate into the host genome, such as HIV (an RNA retrovirus) [2].

Herpesviruses are a family of icosahedral enveloped viruses characterized by a linear double-stranded DNA genome ranging in size from approximately 150 to 250 kb with terminal repeats at each end of the DNA. After infection, the virus undergoes uncoating, and cell-free DNA becomes circularized thanks to terminal repeats enabling the virus to persist in the nucleus as an episome. Notably, viral DNA in the infectious virion is not “chromatinized” yet, but chromatinization occurs during episome formation. The term “chromatinized” refers to a DNA sequence or region of the genome that has been incorporated into the structure of chromatin, the complex scaffolding of DNA and proteins (primarily histones) that forms chromosomes in eukaryotic cells. When a replication origin is chromatinized, it means it is surrounded and influenced by nucleosomes, which can reduce the mobility of key proteins and constrain their position, thus affecting the efficiency of DNA replication [1].

**Figure 1:**
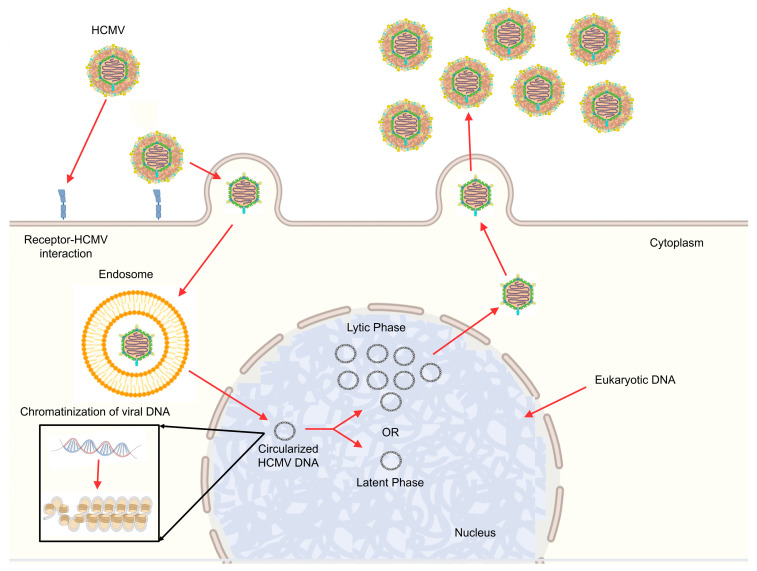
HCMV life cycle. HCMV viral infection is a complex, sequential process involving several phases. HCMV enters the cell through membrane fusion or receptor-mediated endocytosis. Subsequently, the viral genome is transferred to the nucleus and chromatinized, leading to the expression of time-regulated genes. This is followed by capsid assembly, acquisition of membrane proteins, packaging into lipid bilayers, and, finally, the release of new viral particles (lytic phase), or the establishment of the latent phase, where no new virions are released and the HCMV DNA resides in the nucleus as an episome (Some parts of the figure were created in BioRender. Biorender5, U. (2025) (Lab–Academic). https://BioRender.com/u75ciss; https://BioRender.com/3f6rhgx).

The episome can exist as a fully functional virus, capable of replicating and generating new virions, or it can exist in a latent form in which only a limited number of viral control elements are produced without replication or subsequent virion production. Since cells can be lytically infected or latently infected with subsequent reactivation of the virus, great interest has been raised in studying the role played by epigenetics in controlling these biological processes (Figure 1) [2].

## 4. Human Cytomegalovirus: General Characteristics

Herpesviruses are divided into three subfamilies (alpha, beta, and gamma), which differ primarily in their replication characteristics, tropisms (preferred tissues), and infection outcome. Alpha-herpesviruses, such as herpes simplex virus (HSV-1 and HSV-2) and varicella zoster virus (VZV), have a rapid life cycle and tend to cause infections that remain latent in nerve ganglia. Beta-herpesviruses, such as cytomegalovirus (CMV) and roseola virus, replicate more slowly and infect a variety of tissues, including salivary glands and immune cells. Gamma-herpesviruses, such as the Epstein–Barr virus (EBV) and Kaposi’s sarcoma-associated herpesvirus (KSHV), are associated with the onset of tumors and predominantly infect lymphocytes [30,31,32].

Human cytomegalovirus (HCMV) is a member of the beta-herpesvirus family (Human Herpes Virus 5 (HHV5)) that causes significant morbidity in immunocompromised hosts.

HCMV is a ubiquitous infectious agent that causes lifelong infections in humans with variable severity of symptoms based on the immunological status of the infected individual. In immunocompetent individuals, HCMV infection presents a minimal subclinical risk, whereas infection in immunocompromised hosts, such as immunosuppressed recipients of solid organ or bone marrow transplants, is associated with an increased risk of acute and chronic allograft rejection, infection by other opportunistic pathogens, graft failure, and death [33,34].

HCMV has a high seroprevalence globally and in all socioeconomic levels, whereas its distribution is inversely correlated to the socioeconomic development of the region or country, with a prevalence ranging from 44 to 96% [35].

Effective antiviral drugs have reduced the incidence of post-transplant complications due to CMV infection. Antiviral drugs for HCMV include ganciclovir and its oral prodrug valganciclovir (first-line treatment), as well as other agents like foscarnet and cidofovir, which are used for ganciclovir-resistant CMV or in cases of severe side effects. Maribavir is approved for refractory CMV disease in transplant recipients, particularly in those with resistance to traditional agents [36]. However, in many cases, these drugs have only delayed disease onset, and their use is constrained by toxicity and the selection of resistant strains [37].

CMV spreads through direct exposure to various human bodily fluids, including oropharyngeal secretions, urine, semen, breast milk, tears, and blood, as well as cervical and vaginal secretions. Humans are the main natural reservoir.

Congenital HCMV (cHCMV) is caused by vertical mother-to-fetus transmission of the virus via the hematogenous route through the placenta by infected leukocytes [38]. cHCMV infection can be either asymptomatic or symptomatic with severe manifestations, resulting in permanent sequelae with characteristic clinical manifestations such as low birth weight, jaundice, hepatosplenomegaly, microcephaly, intracranial calcifications, purpuric rash, thrombocytopenia, chorioretinitis, mental and motor retardation, epilepsy, neurosensory deficits (e.g., sensorineural hearing loss (SNHL)), hemolytic anemia, and sometimes even death [39,40].

## 5. HCMV Lifecycle

Unlike alpha or gamma herpesviruses, which have a narrow cellular tropism, CMV has a broad tropism (epithelial, endothelial, smooth muscle and connective tissue cells, as well as specialized parenchymal cells in various organs) [41]. Like all herpesviruses, HCMV has the ability to establish a permanent latent infection, in which viral DNA remains present in the body but is undetectable by standard tests because it is in a nonreproductive, quiescent state but is capable of reactivation from latency under appropriate conditions. The alternation between the latency and lytic (reactivation) phases of HCMV has been challenging due to its strict species specificity. Fortunately, murine cytomegalovirus (MCMV) constitutes a useful model for exploring various aspects of HCMV pathogenesis [42].

Viral infection is a complex process, tightly regulated by both viral and host factors. Immediately after infection, based on a dynamic balance between viral factors and cellular responses, the virus can enter one of two phases: (i) the active lytic phase or (ii) the latent phase. A discriminant between the two phases is mainly given by the type of cell infected. Infection of permissive cells such as epithelial, endothelial, fibroblastic, or macrophage cells typically results in lytic viral replication. In these cases, viral transcription is robust, leading to the generation of countless viral genomes that become encapsulated within nascent infectious particles (virions). Whereas, when newly infectious virions encounter undifferentiated cells of the myeloid lineage, such as CD34+ hematopoietic progenitor cells (HPCs), the resulting infection is latency, during which viral gene transcription is largely silenced, with the concomitant absence of viral genome replication and production of infectious viral particles [3,4]. Primary infection of CD14+ monocytes results in a subclassification of latency, defined as “quiescence” [43,44]. This phase, like the latent phase, is defined by the absence of viral lytic replication, associated with limited transcription [3,4]. The fundamental difference between quiescence and latency is that infected CD14+ monocytes undergo an extension of their average lifespan, maintaining their state for a longer time, associated with improved migration and a greater propensity to differentiate into replication-permissive macrophages [45], thereby facilitating viral dissemination to tissues such as the bone marrow [44]. Furthermore, both quiescent and latent remain capable of viral reactivation if subjected to appropriate conditions, including myeloid cell differentiation into dendritic cells or macrophages, as well as stress stimuli such as hypoxia or inflammation [3,46], thus removing a cell type- or differentiation-specific block.

### HCMV Lifecycle: Gene Regulatory Network in the Lytic Phase

**Figure 2:**
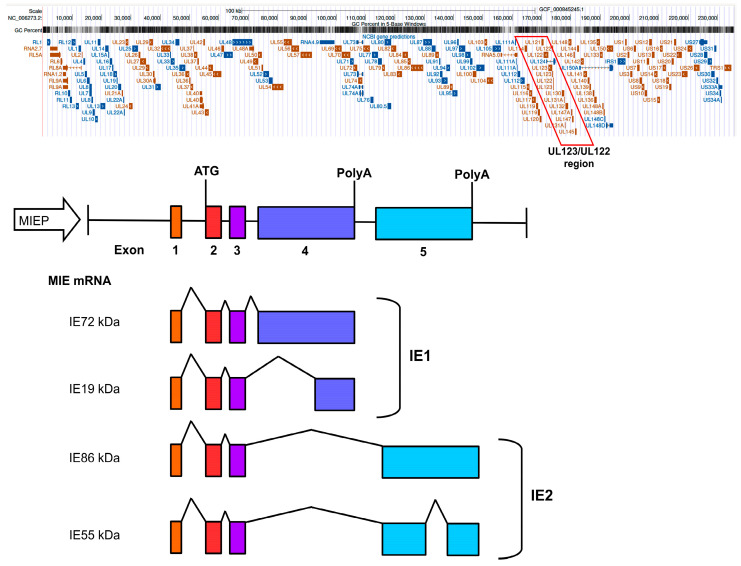
Structural organization and protein products of the major CMV IE locus. The upper part of the figure shows the entire HCMV genome, highlighting (red box) the critical locus encoding the viral proteins immediate-early 1 (IE1) and immediate-early 2 (IE2), essential for viral replication and host cell manipulation (UL122/UL123 region). The lower part of the figure shows the lengths and relative positions of exons 1 to 5 (four coding exons (exon 2 to exon 5), while exon 1 is non-coding), and the position of the major IE promoter-enhancer (MIEP). The resulting proteins are divided into the IE1 (containing the exon 4 sequence) and IE2 (containing the exon 5 sequence).

Herpesviruses, including HCMV, follow a precise temporal sequence of gene expression during a productive lytic infection [5]. This process is temporally divided into three hypothetical phases: (i) immediate early genes (IEgs); (ii) early genes (Egs); and (iii) late genes (Lgs). Initially, IEgs are transcribed, driven by activation of the major immediate early locus (MIE), composed of the core promoter and proximal and distal enhancers. The MIE is a crucial regulatory region of HCMV that drives the expression of IEgs. These IEgs are among the first to be transcribed during viral infection and are essential for the initiation of productive lytic replication. MIE is also a key target for regulating viral latency, as it is silenced during the onset of latency and must be reactivated for the virus to re-enter the lytic cycle. This region also features several binding sites for transcription factors [47], whose association with this locus ultimately controls the expression of the two canonical MIE products, IE1 (IE72) and IE2 (IE86), encoded by UL123/UL122, respectively. These two genes arise from differential splicing of the initial transcript, producing multiple RNAs (Figure 2). Although several protein products expressed from these mRNAs (splice variants) have been identified [40,48], the 72 kDa nuclear phosphoproteins IE1 (IE72) and IE2 (IE86) are the most abundant and of greatest interest (Figure 2). They share 85 amino-terminal amino acid residues, corresponding to IE exons 2 and 3, but distinct carboxy-terminal portions, encoded by exon 4 (IE1) or exon 5 (IE2), respectively (Figure 2). While IE2 is essential for productive viral replication [49], IE1 is only conditionally essential. Indeed, experiments conducted with viruses lacking IE1 showed univariate replication efficiency in fibroblasts at high multiplicity of infection (MOI) but exhibited severely attenuated replication under low MOI conditions. Concomitantly, the absence of IE1 results in severely attenuated viral replication under low MOI conditions [50,51,52,53]. Other transcripts and gene products derived from alternative promoters within the intergenic and intronic sequences also play a role in HCMV infection depending on the type of infected cells and their differentiation state (Figure 2) [54,55,56]. Once IE72, IE86, and the other IE proteins are expressed, incremental progression of the viral transcriptional cascade continues through the initiation of synthesis of Eg products, triggering viral DNA replication. Finally, Lg are expressed, which encode the structural proteins of the virion, such as those of the capsid and the pericapsid, as well as those required for the assembly and release of new virions. In summary, HCMV, during lytic infection, activates a cascade of gene expression that leads to the production of specific proteins at each stage, ensuring the replication and dissemination of the virus [57,58,59,60].

As infection progresses, the viral protein IE86, in a typical negative feedback mechanism, binds to its own promoter, inhibiting it, facilitating the repression of the MIE promoter (MIEP) [61,62]. Furthermore, IE86 has been reported to transactivate specific early and late gene promoters [58], which interact with nucleosomes on viral chromatin [57]. During the latency phase, repression of the MIE locus silences transcription from this region as well as from downstream genes required for efficient viral DNA replication [5,63].

## 6. Chromatinization of HCMV Genome

The progression of viral transcription is strongly influenced by the environment of the infected host cells and changes based on their responses to internal/external stimuli or their state of differentiation. Once HCMV enters and infects cells, the virus manipulates available host factors through the MIEP, which in turn induces IEg. The environment, for example, of a differentiated macrophage differs greatly from the environment of a hematopoietic progenitor cell (HPC), thus influencing the progression towards a lytic infection in macrophages or a latent infection in HPCs. For an effective utilization of the host factors, the viral genome must be both accessible to these factors and sufficiently stable to persist without triggering the host’s innate responses.

There are several mechanisms that allow viruses to escape the host’s innate immune system. HCMV achieves this through chromatinization of its genome, following the translocation of herpesvirus genomes into the cell nucleus, where host-derived histones rapidly associate with the viral genome to form stable nucleosomes [64,65,66], which, in the case of HCMV, occurs within 30 min of infection [67].

Ultimately, it can be assumed that, initially, the association of histones with the HCMV genome is largely determined by the viral DNA itself, based on intrinsic histone sequence preferences [66], and then evolves towards a lytic or latent state depending on the infected cell line.

### 6.1. Anchoring Sites of Viral Genomes to Host Chromosomes

Once the viral genome of herpesviruses has penetrated the host cell nucleus, within a few minutes, it undergoes a series of conformational changes such as the loss of the viral capsid (uncoating), chromatinization, circularization and anchoring of the genome to the host chromosomes, giving it stability and functionality. Unlike the genomes of gamma-herpesviruses, which bind to host chromosomes during the latency phase via viral proteins, such as Epstein–Barr nuclear antigen 1 (EBNA1) for Epstein–Barr virus (EBV) [60,61,68,69], or in the case of Kaposi’s sarcoma-associated herpesvirus (KSHV) via latency-associated nuclear antigen (LANA) [70], an HCMV-encoded anchoring protein that functions during latency has not yet been identified.

A possible candidate for such a role is the viral protein IE72, which contains a chromatin tethering domain (CTD) able to interact indirectly, via the cellular Specificity Protein 1 (SP1), with histones [71]. SP1 displays tissue-specific expression profiles [72] and is upregulated during lytic infection [73]. The chromosomal association was initially mapped approximately to the MIE exon 4 sequences (Figure 2) [66,74] and was subsequently narrowed down to residues 421 to 486 of the 491-amino acid viral protein (exon 4) [75]. Finally, it was established that the 16 carboxy-terminal residues (amino acids 476 to 491) of IE1 were necessary and sufficient for interaction with mitotic chromatin in transfected cells and were consequently named “chromatin anchoring domain” (CTD) [76]. Chromosome anchoring capacity appears to be evolutionarily conserved among primate CMV IE1 orthologs [77,78]. However, despite being a conserved and distinctive feature of IE1, the mechanisms underlying chromosomal association mechanisms by the viral protein remain undefined.

Complicating matters, Mauch-Mucke et al. recently found that HCMV genomes bind to host mitotic chromosomes in both non-permissive KG-1 myeloid cells and permissive fibroblasts, whereas in the latter this process occurs in an IE72 (IE1)-independent manner [79], suggesting that some other viral protein may be involved in anchoring the HCMV genome to cellular chromatin. As an alternative anchoring candidate, IE19, a lytically expressed protein, possesses its own CTD that helps retain the viral genome in the nucleus during mitosis, before cells enter the G1 phase in permissive fibroblasts [80]. Indeed, this domain corresponds to the carboxy-terminal part of exon 4 of IE1 (see Figure 2), so the CTD would appear to be the same as that present in IE72. Unfortunately, to date, no anchoring function has been documented that brings the viral genome near to the host nuclear machinery necessary to modulate the host epigenetic environment and influence the recruitment of the transcriptional machinery.

### 6.2. Nucleosome Formation in HCMV DNA as a Cellular Repressor Mechanism

**Figure 3:**
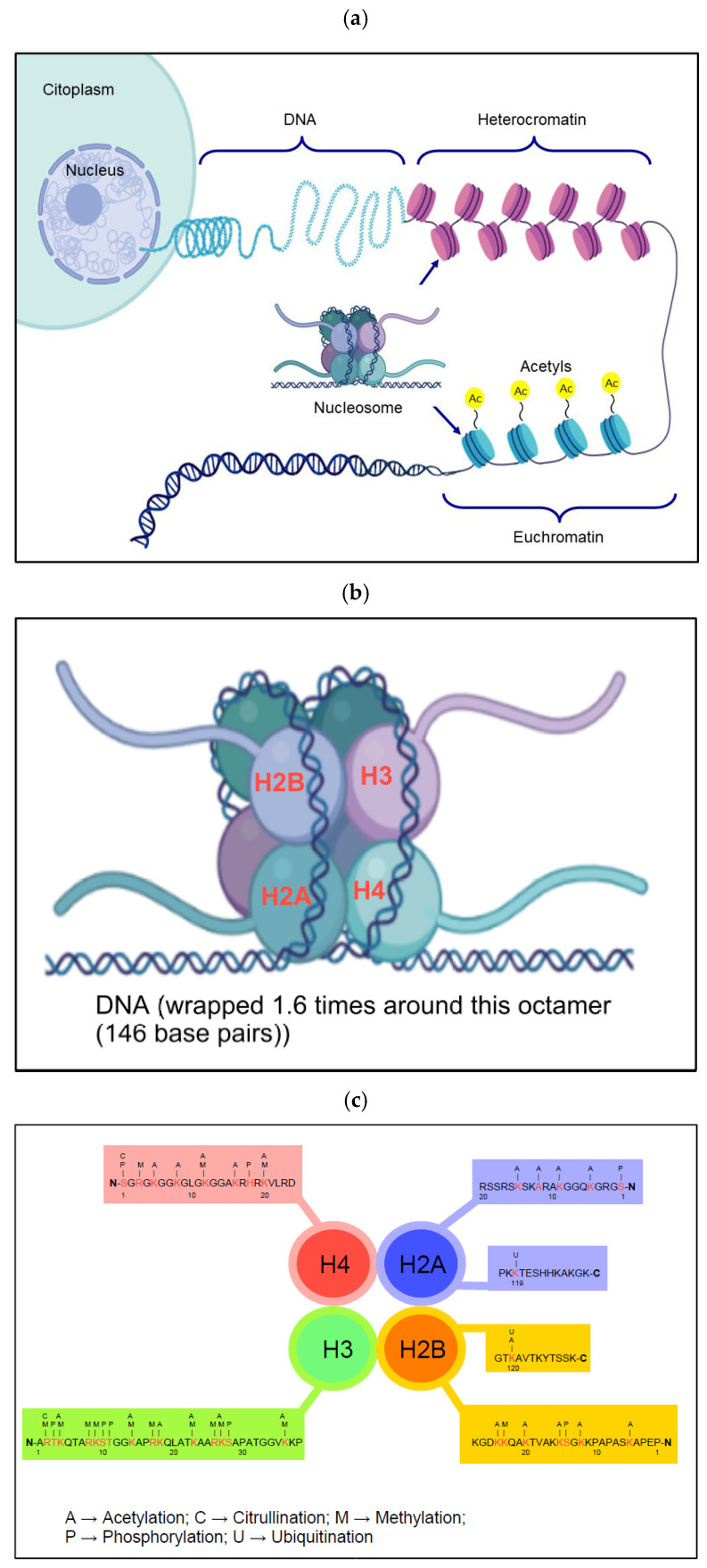
(**a**) Summary figure on the distinction between hetero- and euchromatin (Figure drawn using the “Biorender” software). (**b**) Detail of the nucleosome, consisting of a stretch of DNA wrapped around a protein core called the histone core. The histone core is composed of eight histone proteins: two copies each of the H2A, H2B, H3, and H4 types (Some parts of the figure were created in BioRender. Biorender5, U. (2025) (Lab–Academic). https://BioRender.com/anc033v; https://BioRender.com/lgvpdcg). (**c**) Schematic representation of the main PTMs of the N- and C-terminal ends of the four histone types present in the nucleosome.

Highly condensed chromatin, or heterochromatin, is a compact and dense form of chromatin that limits access to macromolecules, rendering the region inactive for DNA replication, transcription, DNA damage repair, and recombination. This state, however, is reversible, thanks to numerous time- and space-dependent biological processes that allow the reactivation of various previously inactivated biological functions (Figure 3a).

Nucleosome formation in mammalian cells is a dynamic and highly efficient process that occurs primarily at replication forks during S phase, either by the deposition of histones from the parental strand or by de novo assembly into the complementary strand [81]. Nucleosome assembly proceeds in a two-step process that begins first with the assembly of a tetramer consisting of two H3 and two H4 histone subunits, followed by a second tetramer composed of two H2A and two H2B subunits. Finally, the DNA is wrapped 1.6 times around this octamer (146 base pairs) (Figure 3b) [82]. Similarly, during lytic infection, the double-stranded HCMV genome is replicated by replication-dependent nucleosome assembly [64]. Initial deposition of histones on the HCMV genome occurs rapidly after entry into the nucleus within approximately 30 min. At this stage, the polymerase has been shown to be inhibited [64], suggesting that the process of chromatin formation precedes both transcription and new viral replication [67]. Initially, nucleosome formation occurs with the deposition of octamers at GC-rich viral loci by host cell proteins, while with the initiation of viral IEg transcription, chromatin formation is directly driven by the viral IE72 protein [66], which associates with the acidic region of the H2A-H2B tetramer via the cellular protein SP1 on the surface of nucleosomes [83,84]. Thus, the gradual increase in histone occupancy occurs simultaneously with viral DNA synthesis, with histones becoming methylated at the H4K4 level (an epigenetic modification that, in general, is associated with the activation of gene expression) [64,85]. Therefore, it can be stated that chromatinization of the HCMV genome appears to be independent of the cell cycle of the infected host.

In viral chromatin, as well as in cellular chromatin, all four classes of histones and their different isoforms are present (see above) [86]. The inclusion in the chromatin of the non-canonical variant of the H3 subunit, “H3.3”, is of particular interest in the context of productive infection, as it is incorporated into nucleosomes at specific genomic loci, indicating that this variant performs distinct functions [87,88].

The addition, exchange, deposition, and expulsion of histones occur in DNA through a continuous series of nucleosome assembly and disassembly, mediated by specific and highly efficient histone chaperones.

Two histone chaperones involved in the recognition and regulated deposition of H3.3 through the replication-independent pathway are histone cell cycle regulator A (HIRA) and death domain-associated protein (DAXX), in collaboration with the chaperone complex component, the alpha-thalassemia/mental retardation-associated X-linked protein (ATRX). The “Daxx/ATRX” complex plays a crucial role in maintaining HCMV chromatin structure by binding specifically to the histone variant H3.3, whereas these two proteins act as molecular chaperones, transporting H3.3 for deposition in specific regions of the genome. This interaction is important for gene silencing and genomic stability [87]. The interaction of H3.3 with Daxx/ATRX represses HCMV transcription very rapidly after infection of permissive cells, an effect that is attenuated by Daxx inhibition [89]. Indeed, studies on the multiplicity of infection (MOI), have shown that the higher the initial viral load (higher MOI) the easier the Daxx-induced repression in the early phase of lytic infection is overcome [89]. HIRA, a chaperone protein that deposits H3.3 onto foreign DNA, cooperates with ATRX/Daxx to repress foreign DNA. Indeed, mice infected with murine CMV (MCMV) lacking HIRA show substantially higher viral loads [90].

## 7. Overcoming Repression of the HCMV Genome

The cellular structures where ATRX/Daxx and HIRA localize to repress foreign DNA are known as nuclear domain-10 (ND-10) or promyelocytic leukemia protein (PML) nuclear bodies (PML-NB). PML-NBs are dynamic nuclear structures composed of PML and non-PML structural proteins (Daxx, Sp100, ATRX, HDAC, HP1, SUMO-1, etc.) important for the regulation of genome organization and involved in several cellular processes such as DNA damage response, DNA repair, response to viral infection, apoptosis, protein modification, transcriptional regulation, cell proliferation, senescence and tumor suppression [83,84,91,92,93]. These complexes are believed to repress viral gene expression as part of an intrinsic host immune response [92].

An early cellular response to viral infections, including HCMV ones, has been associated, by numerous studies, with the recruitment of heterochromatin protein 1 (HP1) by PML-NB nuclear bodies, acting as a first barrier to the initial stages of viral infection [94,95,96].

To overcome this initial block and activate viral gene expression, HCMV has evolved some very ingenious solutions, such as proteins present in the viral tegument that enter the cell following fusion of the viral and host membranes and immediate-early proteins, which are transcribed early in the infection. Furthermore, viral chromatin undergoes dynamic changes during infection, such that, very early after the onset of infection, histones bound to HCMV promoters exhibit modifications consistent with transcriptional repression, which are gradually replaced by euchromatic modifications as “immediate-early” gene expression is activated [67,92]. Thus, HCMV antagonizes PML-NB function through the viral tegument protein pp71, which is involved in the activation of immediate-early viral gene expression, regulation of cell cycle progression, and immune system evasion. Once the virus lytically infects epithelial, endothelial, fibroblastic or macrophage cells, pp71 translocates to the nucleus, blocking the main repressive components of PML-NB and inducing the degradation of Daxx. This interaction is crucial to increase the infectious capacity of viral DNA, thus allowing transcription of the viral genome [97]. However, following latent infection of CD34+ pluripotent stem cells (HPCs), pp71 is retained in the cytoplasm and thus fails to disrupt PML-NB-induced defenses [98], suggesting cell type and/or infection stage specificity. Other viral proteins capable of blocking PML-NB have been studied in recent years, including UL35 [99], UL97 [100], Latency Unique Natural Antigen (LUNA) [101,102], IE72 [77,101,102,103,104], and IE86 [105]. E.g., IE72 counteracts Daxx-induced repression through its binding to HDAC3 in lytically infected cells by blocking histone deacetylation [106]. Indeed, various histone deacetylase inhibitors (HDACi) have been shown to increase the efficacy of HCMV and MCMV infection in human and mouse cells, respectively, indicating that HDACs repress viral DNA replication at the transcriptional level through increased chromatin compaction and subsequent gene silencing [67,107]. As lytic infection progresses, IE72 directly interacts with Daxx, disrupting the Daxx/ATRX complex and promoting viral transcription [108]. Furthermore, IE72 blocks SUMOylation of the structural component of PML-NBs, compromising their integrity [104]. SUMOylation is defined as a post-translational modification that involves the enzymatic addition of small ubiquitin-related modifier (SUMO) to proteins, which regulates protein-protein and protein-DNA interactions and promotes protein solubility [109]. Indeed, IE72 indirectly influences PML stability, since IE72-Daxx interaction drives LUNA transcription during lytic infection [108]. LUNA is a cysteine protease with deSUMOylase activity. Interestingly, the viral LUNA gene product was found to be expressed during both latent and lytic infection [110], as well as during reactivation from the latent to the lytic phase in monocytes [111]. Unfortunately, the mechanism remains unclear to date. Transfection experiments of wild-type LUNA viral protein into HCMV-infected cells promoted the destruction of PML-NB complexes. Although the LUNA protein was not required for the latent phase, a significant defect in reactivation (latent/lytic phase transition) was observed in experiments with LUNA-null proteins or a catalytically inert version of LUNA. Furthermore, elimination of PML from cells restored the reactivation capacity of latent viruses with defective or missing LUNA proteins, confirming the biological importance of PML deSUMOylation by LUNA. This viral activity is critical during the very early stages of HCMV reactivation, leading to a robust induction of IE gene expression, with downstream consequences for viral reactivation [101]. Data reported so far suggest that PML-NB subnuclear structures contribute to the establishment and maintenance of heterochromatin, influencing multiple aspects of HCMV transcriptional control.

Once repression by PML-NBs through the above-mentioned mechanisms is overcome, the viral genome must maintain a euchromatic structure that is more accessible to the enzymes involved in gene transcription. In general, this is achieved through specific epigenetic modifications, mainly related to chromatin and DNA through post-translational modifications (PTMs) of histone surfaces and their unstructured tails.

PTMs are chemical modifications of proteins, and the enzymes that regulate them are classified as “writers”, “readers” or “erasers” [112].

Writers catalyze the addition of chemical groups to proteins, such as acetylation or methylation. For example, histone acetyltransferases (HATs) add acetyl groups to histones, while histone methyltransferases (HMTs) add methyl groups. Readers are proteins that bind to modified amino acid residues on proteins, acting as interpreters of the PTM code. They recognize specific modifications and recruit other proteins or influence their activity, ultimately dictating the cellular response (For example, methyl-binding protein, bromodomains, tudor domains, and chromodomains). Finally, erasers are enzymes that remove these modifications. Histone deacetylases (HDACs) remove acetyl groups, while histone demethylases (HDMs) remove methyl groups. Together, these PTMs directly or indirectly influence target gene expression by altering DNA accessibility or by recruiting cofactors to induce these changes and remodel surrounding nucleosomes. For example, acetylation of lysine 27 of histone 3 (H3K27ac) is associated with transcription activation and is used as a marker for active enhancers, distinguishing them from inactive or “waiting” ones, while trimethylation of the same H3K27 residue (H3K27me3) is associated with global gene repression, leading to the formation of heterochromatic regions and gene suppression. H3K9 trimethylation (H3K9me3) is also associated with heterochromatin formation and transcriptional silencing [85].

Some epigenetic markers also demarcate specific chromatin regions, such as H3K4me3, which is found in the gene promoter, indicating that the gene is open and ready to be transcribed, while H3K4me1 and H3K27ac are associated with potentially active enhancers [113,114].

## 8. Reversibility of the HCMV Genome from the Latent to the Lytic State

### 8.1. Latent Infection (Constitutive Heterochromatin)

After an initial lytic phase in permissive cells (epithelial cells, endothelial cells, fibroblasts, macrophages, etc.), in which a large number of virions are produced that infect adjacent cells, including non-permissive cells entering a latent phase (undifferentiated cells of the myeloid lineage, such as hematopoietic progenitor cells (CD34+)), the viral genome becomes largely heterochromatinized, inaccessible to transcription factors thanks to the recruitment of corepressor complexes, composed of enzymes that catalyze repressive histone modifications, compact chromatin, and prevent nucleosome remodeling [115,116].

Unfortunately, to date, our knowledge of the basic mechanisms for maintaining viral latency remains incomplete and fragmented despite numerous studies and some significant progress made. For example, herpes simplex virus-1 (HSV-1) maintains latency through Latency-Associated Transcripts (LAT) [117], while in the case of gamma-herpesviruses, such as the Epstein–Barr virus (EBV), key components like Epstein–Barr virus-encoded small RNAs (EBERs) and Epstein–Barr nuclear antigens (EBNAs) play crucial roles in the viral life cycle and impact on the host cell [118].

The most important regulatory region of the HCMV genome in the context of epigenetic regulation of latency is the MIE locus. The DNA of the MIE locus in a non-permissive cell is characterized by various histone PTMs (acetylation, methylation, phosphorylation, ubiquitination, lipidation, etc.) associated with heterochromatin (facultative or constitutive) and histone methylation-induced transcriptional repression, such as H3K9me2, H3K9me3, and H3K27me3, which influence gene activity (Table 2 and Figure 3c) [6,119,120,121,122,123,124,125,126,127,128,129,130,131,132,133,134,135,136,137]. Other, non-epigenetic factors regulate the HCMV life cycle, such as the transcriptional corepressor, Krüpple-associated protein 1 (KAP1/TRIM28), which localizes to nuclear foci in close spatial proximity to NB-PMLs [138]. KAP1 binds to Krüpple-associated box (KRAB) domains on DNA-bound KRAB zinc fingers (ZNFs), recruiting SETDB1 (SET domain bifurcated histone lysine methyltransferase 1) to facilitate H3K9 methylation [139,140], resulting in transcriptional repression. Indeed, it has recently been shown that KAP1 is recruited to the HCMV genome through host factors such as SERPINE mRNA-binding protein 1 (SERBP1) and chromodomain helicase DNA-binding protein 3 (CHD3). These interactions serve to link SERBP1 to the chromatin remodeling function of CHD3, suggesting a role in gene regulation, thereby influencing mRNA stability and translation as part of the nuclear NuRD (Nucleosome Remodeling and Deacetylase) complex, inducing transcriptional silencing [141]. KAP1 also binds HP1, further promoting H3K9 methylation and consequently heterochromatin expansion [142]. Rapamycin studies have shown that its use induces indirect phosphorylation of KAP1 through its desumoylation by mTOR and subsequent phosphorylation at the same sites. Phosphorylated KAP1 reduces its association with HP1 and SETDB1 and, in turn, facilitates the transcription of lytic genes [138]. Overall, these findings highlight the multiple mechanisms through which HCMV latency is maintained. A full understanding of these factors remains still elusive, although it is clear that repressive epigenetic modifications and host proteins that contribute to transcriptional silencing are central to maintaining this state of infection.

### 8.2. Latent Infection (Facultative Chromatin)

Facultative heterochromatin (more than constitutive heterochromatin) has been shown to be very important for productive HCMV infection due to its propensity to change its state of condensation and transcriptional activity depending on the cell type or developmental stage, influencing the expression of specific genes. Indeed, infection of permissive mouse cells with MCMV has been shown to increase H3K27me3, a typical marker of facultative heterochromatin in the MIE region. Subsequently, within 3 h of infection, the methylation of these residues is replaced by increasing levels of acetylation, resulting in chromatin opening (Table 2 and Figure 3c) [143]. It was subsequently shown that the intrinsic pre-IEg viral response is also found in human CMV [67]. The H3K27 demethylase, namely lysine demethylase 6B (KDM6B/JMJD3), is inhibited by pUL138 produced from the viral UL138 gene, which is required for maintaining latency in CD34^+^ HPCs [144]. UL138 is part of the UL133-138 locus, a region of the viral genome that is crucial for the regulation of latency and reactivation. If not inhibited, KDM6B localizes to and derepresses MIEP through the removal of H3K27 methylation [7]. All this suggests an active role of the Polycomb Repressor Complex (PRC), PRC2 and PRC1, constituted by Polycomb-group proteins (PcGs) that play a crucial role in regulating gene expression and maintaining gene expression patterns during cell development and differentiation. These complexes act by modifying chromatin, making it less accessible to the transcriptional machinery and suppressing gene activity. The two complexes interact with each other, with PRC2 recruiting PRC1 to H3K27me3 methylation sites. In particular, EED, a PRC2 protein, can interact with PRC1 and enhance its ubiquitination activity. Thus, PRC2 establishes the basis for repression, while PRC1 maintains the stability of this state. Indeed, they act in concert as “writers”, whereas PRC2 introduces mono-, di-, and tri-methyl modifications on H3K27, and PRC1 mediates the ubiquitination of H2AK119 via H2A ubiquitination at lysine 119 (H2AK119ub), which is a crucial epigenetic marker mainly associated with gene repression through PRC1. This process, where ubiquitin molecules are bound to lysine 119 of histone H2A, influences chromatin structure and regulates gene expression (Table 2 and Figure 3c) [145]. Indeed, different studies have shown that PRC1 and PRC2 contribute to reducing productive infection [145,146]. However, to date, no direct association between PRC complexes and the HCMV genome has been identified. Recently, it was discovered that the subunits EZH2 (enhancer of zeste 2 polycomb repressive complex 2 subunit) and BMI1 (proto-oncogene BMI1, polycomb ring finger) coprecipitate with the viral lncRNA RNA4.9, also precipitating the actively replicating HCMV viral genome. This occurs because RNA4.9, transcribed from the HCMV origin of replication (oriLyt), forms an RNA-DNA hybrid (R-loop) through its G+C-rich 5′ end [7,145,147]. While EZH2 and BMI1 are both Polycomb protein cluster rearrangement factors, RNA4.9 plays a crucial role in regulating viral DNA replication. During this phase, RNA4.9 binds to the MIEP during latency, concomitantly with the appearance of H3K27me3, suggesting that RNA4.9 and PRC2 support the repression of this promoter [8,143] and indicating a direct role for facultative heterochromatin in the control of viral transcription. Finally, in support of this, it was shown that HIRA-mediated H3.3 deposition recruits PRC2 in MCMV-infected murine cells [148]. Overall, these data suggest that PRC complexes are involved in the repression of neochromatinized CMV genomes both during initial infection and during the latency phase.

### 8.3. Lytic Replication and Reactivation

The lytic state of HCMV, which occurs either after the first infection of permissive cells (primary infection) or after reactivation from the latent stage (secondary infection or reactivation), shares a very similar gene expression profile, with the production of virions in host cells, cell lysis, and infection of neighboring cells with the potential to infect other individuals.

The resulting change in the recruitment of chromatin-associated proteins and transcription factors, such as those regulating histone PTMs, alters the balance of the cellular and viral environment, helping to drive cellular differentiation on the one hand and the viral switch with viral lytic reactivation on the other. The elements that characterize this process are the acetylation of H3K9 (H3K9ac), the phosphorylation of H3K10 (H3K10p) mediated by cAMP responsive element binding protein (CREB), the loss of the characteristic binding of HP1 with PML-NBs, as well as the concomitant loss of methylation in di- and tri-methylated H3K9 at MIEP (Table 2 and Figure 3c) [107,149,150]. The result of these epigenetic changes, ultimately, corresponds to the contextual opening of the viral chromatin in proximity of the lytic gene promoters, which in turn become accessible to the transcription factors and enzymes necessary for the replication and transcription of the lytic genes, Eg before and Lg after [67], with a concomitant increase in IEg transcripts (IE72 and IE86) and a reduction in typical repressive markers [67]. Furthermore, both IE72 and IE86 bind HDAC3, inducing the opening of condensed chromatin and subsequent transcription of silenced genes [106], recruiting polymerase II and promoting H3K27 acetylation (H3K27ac) on the OriLyt RNA4.9 promoter in infected fibroblasts [151]. OriLyt, an acronym of “origin of lytic replication,” is a specific DNA sequence essential for the replication of some viruses during their lytic cycle. Since OriLyt is a bidirectional promoter [152], it has been hypothesized, but not yet proven, that this region functions as an enhancer element to influence the expression of the E gene [151]. Overall, these data support a model that illustrates a complex series of transcriptional changes in response to changes in the surrounding epigenetic landscape.

## 9. Future Prospects: Epigenetic Reprogramming of Viruses

The relationship between HCMV and epigenetics is a rapidly expanding new field of research that is developing on two parallel tracks: on the one hand, understanding the basic mechanisms by which the virus interacts with its host, taking over and controlling the main epigenetic machinery to evade the immune system and to promote viral replication or, alternatively, the latent phase (as seen above); on the other hand, the development of innovative therapeutic strategies: (i) new targets: identification of new epigenetic “targets”; and (ii) new drugs: development of epigenetic drugs that can block epigenetic alterations induced by the virus, thus counteracting the infection (HDACi, DNMTi, HMTi, etc.).

The combination of novel targets coupled with the development of new epigenetic compounds has produced countless therapeutic strategies, the most promising of which are the “shock and kill” and “block and lock” methods, based on the induction or repression of the latent/lytic phase switch, respectively, through the use of epigenetic inhibitors.

The two methods arose from experimental studies on the epigenetic reprogramming of the human immunodeficiency virus (HIV) to control the expression or repression of viral genes. These studies have recently been translated to other viruses, including HCMV, hepatitis B and C viruses, herpes simplex virus, and Epstein–Barr virus. Reactivation of HCMV from the latent phase (“shock and kill” paradigm) or alternatively repression of the lytic phase and thus reactivation of the virus (“block and lock” paradigm) by epigenetic targeted therapy represent encouraging options for viral manipulation [9], representing a pioneering area of research in modern biology that could constitute a curative methodology, potentially allowing the development of new and innovative broad-spectrum antivirals with “epigenetic” action for the control of viral infections in vivo.

Although the few antiviral drugs available to date have allowed important progress in the treatment and prophylaxis of HCMV infection, such as ganciclovir and its oral prodrug valganciclovir, cidofovir, foscavir (foscarnet), fomivirsen [153], and letermovir [154], all approved by the FDA, their clinical applicability faces several obstacles [155]: (i) drug resistance [156]; (ii) poor oral bioavailability; (iii) hematological and renal toxicity [157]; (iv) no effect on the latent viral form of the virus, allowing spread and transmission from body fluids (saliva, urine, milk, vaginal secretions, semen, etc.) [158], being able to act only on viruses in the active phase of DNA replication [159].

Furthermore, one of the major problems attributed to HCMV infection is the post-transplant complications associated with an increased risk of rejection and other serious pathologies such as retinitis or pneumonia attributed to the reactivation of the lytic phase of the virus (viral rebound) [9,160]. This is due to the presence of HCMV reservoirs in hematopoietic lineages such as CD34+ myeloid progenitor cells and their derived CD14+ monocytes. Interestingly, the latent virus within cells is subject to transcriptional silencing by host chromatin-modifying enzymes, including deacetylases, methyltransferases, and others (see above) [161].

To manipulate these chromatin-modifying enzymes, to date, few epigenetic drugs have been approved by the FDA for various diseases (cancer, neurological and psychiatric disorders (Alzheimer’s disease, schizophrenia), autoimmune disorders (rheumatoid arthritis, systemic lupus erythematosus), etc.) [162,163,164], namely, DNA methyltransferase inhibitors (DNMTi) (azacitidine, decitabine) and histone deacetylase inhibitors (HDACi) (vorinostat, romidepsin, belinostat, panobinostat) [165], while other categories of inhibitors are currently under investigation such as histone methyltransferase inhibitors (HMTi) tazemetostat, which acts on the EZH2 enzyme and is used for the treatment of specific solid tumors [166], histone demethylase inhibitors (HDMi) which act on enzymes that remove methyl groups from histones such as LSD1 inhibitors [167], or bromodomain and extraterminal motif protein inhibitors (BETi) that target proteins that “read” epigenetic modifications and bind acetylated histones, influencing gene expression [11,12].

### Lytic Phase Reactivation and Latent Phase Blocking

The “shock and kill” strategy is an experimental approach created to treat HIV and later extended to other viruses including HCMV, which involves two distinct phases: (i) “shock” phase, in which the virus is reactivated from the latent phase characterized by non-replication of viral DNA and non-exposure of viral antigens on the cell surface, making infected cells visible to the immune system or antiviral drugs; (ii) “kill” phase, which consists of eliminating the reactivated cells that display antigens on their cell surface that will be attacked by the immune system (killer or cytotoxic T lymphocytes or CD8+), eliminating the hidden viral reservoir, for which normal anti-retroviral therapies are not effective [168]. This technique is advantaged by the fact that in healthy carriers of the virus, up to 10% of circulating peripheral blood resident cytotoxic T lymphocytes (CTLs) are specific for the HCMV lytic antigen and potently target lytically infected cells [169]; on the other hand, only a limited part of the hidden viral reservoir will be activated by the shock phase [170]. This means that a portion of the viral reservoir remains unchanged, ready to reactivate if treatment is interrupted, preventing a definitive cure and the possibility of transplant rejection.

Of course, most of the data produced so far, as we have said, comes almost entirely from studies on HIV reactivation. Following these studies, in recent years several groups have begun to test different latency reversal agents (LRA) or “shock” inducers [10,171] on cells infected with HCMV/MCMV, including HDACi (vorinostat or suberanilohydroxamic acid (SAHA), Panobinostat, MC1568, etc.) [56], HMTi and DNMTi, inhibitors of BET proteins (GSK726), etc. [11,12].

Another disadvantage of all the treatments discussed above using epigenetic compounds is that there could be large off-target effects by making unwanted and unplanned changes that occur at sites other than the chosen target [168].

The “block and lock” strategy for HCMV involves the use of latency-promoting agents (LPAs) to maintain the virus in a state of deep dormancy, preventing reactivation from its latent reservoir. This strategy differs from the “shock and kill” method and aims to create a “functional cure” by permanently silencing the virus so that it remains suppressed even if antiviral therapy is interrupted. This result is achieved by combining current antiviral therapy with LPAs that suppress viral transcription and induce epigenetic silencing to “lock” the provirus in a state of deep latency [172].

## 10. Conclusions

In recent years, some studies have made clear that epigenetic modifications, which occur in the cellular environment, are very important for the success or failure of a productive viral infection or even, as in the case of herpesviruses, for the switch between the lytic or latent state or, furthermore, for viral reactivation when the immune system is weakened due to disease, immunosuppressive treatments, or transplants.

HCMV gene expression is tightly regulated by the formation of nucleosomes during the chromatinization of viral DNA after it has entered the host cell nucleus [64,67]. The various phases that a herpesvirus can manifest (latency; quiescence; reactivation; lytic infection) are highly complex and dynamically regulated processes, which require a balance between viral and host factors, with the intervention of different types of epigenetic factors, which can be divided into three distinct categories: “writers”, “readers” or “erasers” [112]. These biomolecules finely modulate viral transcription also based on the cellular environment in which they are found.

The initial transcriptional repression induced by viral genome chromatinization as an innate antiviral response is overcome within a few hours to support lytic infection in permissive cells thanks to the presence of specific epigenetic factors, while in non-permissive cells of the myeloid lineage, different epigenetic factors lead to HCMV latency [67,144].

When cells differentiate or are subjected to stress factors (physical, chemical, biological, and environmental) that simultaneously cause a change in their set of epigenetic factors, the virus reactivates and produces infectious particles.

The current body of evidence supports a more effective clinical approach and paves the way for the comprehensive and definitive management of cytomegalovirus (CMV) infection. In particular, novel epigenetic therapeutic approaches, like those termed “shock and kill” and “block and lock”, are under investigation as means to regulate viral latency and reactivation and could be potentially useful for treatment of HCMV in immunosuppressed and/or transplanted patients [9,10,11,12,168,172].

## Figures and Tables

**Table 1:** Presentation of the different HDAC isoforms.

Zn-dependent enzymes	Class	Name	sub-cellular location	Location in body
	Class I (Rpd3 like)	HDAC1	Nucleus	Ubiquitous
		HDAC2		
		HDAC3		
		HDAC8		
	Class IIa (Hda1 like)	HDAC4	Nucleus/Cytoplasm	Tissue specific
		HDAC5		
		HDAC7		
		HDAC9		
	Class IIb (Hda1 like)	HDAC6	Cytoplasm	Tissue specific
		HDAC10		
	Class IV (Rpd3/Hda1 like)	HDAC11	Nucleus/Cytoplasm	Tissue specific
NAD-dependent enzymes	Class III (Sirtuins; Sir2 like)	SIRT (1-7)	Nucleus/Cytoplasm	Ubiquitous: but their expression and activity can vary between different cell types and tissues, influencing the specific biological processes of each tissue.

**Table 2:** Some of the most important epigenetic modifications.

Histone Code	Function	Cites
H3K4me3	Epigenetic modification associated with active gene transcription. It is primarily enriched at transcription start sites (TSSs) and is known to play a role in initiating and regulating gene expression.	[119]
H3K4me2	Histone modification that plays a multifaceted role in gene regulation. It is primarily associated with active gene expression and is often found at promoters and enhancers of actively transcribed genes. However, its function can vary depending on the organism and specific genomic context. In some cases, it can also act as a repressive mark.	[120]
H3K4me	Histone modification that plays a crucial role in regulating gene expression. It is associated with active gene promoters and enhancers, and is involved in various cellular processes, including development, differentiation, and synaptic plasticity.	[121]
H3K36me3	Epigenetic modification that plays a crucial role in various cellular processes, primarily by influencing transcription and DNA damage repair. It acts as a marker on actively transcribed genes, affecting processes like transcription fidelity, mRNA splicing, and DNA repair.	[122]
H3K36me2	Histone modification that plays a multifaceted role in gene regulation and DNA repair. It is involved in restricting the spread of H3K27me3, recruiting the Rpd3S histone deacetylase complex, and contributing to double-strand break repair.	[123]
H3K36me	H3K36me plays a crucial role in maintaining genome stability. It is involved in various cellular processes, including transcription, DNA damage repair, and gene expression regulation. H3K36me modifications are deposited by specific enzymes and recognized by other proteins, influencing chromatin structure and function.	[124]
H3K79me3	Epigenetic mark involved in various cellular processes. It is associated with active transcription, DNA damage repair, and cell cycle regulation. Specifically, H3K79me3 is enriched at actively transcribing genes and is required for the expression of certain genes. It also plays a role in the DNA damage response and cell cycle checkpoint control.	[125]
H3K79me2	H3K79me2 plays a crucial role in regulating gene expression. It is primarily known for its involvement in transcription elongation, where it is associated with faster transcriptional rates. However, it also has roles in other processes like enhancer activity, DNA damage response, and cell fate determination.	[126]
H3K79me	Methylation of H3K79 is involved in the regulation of telomeric silencing, cellular development, cell-cycle checkpoint, DNA repair, and regulation of transcription.	[127].
H3K27me3	Epigenetic modification that generally leads to gene silencing. It is a key player in maintaining stable gene expression patterns, particularly during development and cell differentiation. This modification is associated with inactive gene promoters and is often found in regions where gene expression needs to be suppressed.	[128].
H3K27me2	Epigenetic modification that plays a role in gene regulation. It is often found in broad chromatin domains and is thought to help prevent the unscheduled activation of enhancers. While it is associated with transcriptional repression, it can also be found at active regulatory regions, suggesting a more complex role than simple silencing.	[129]
H3K27me	This modification is primarily associated with gene silencing and plays a crucial role in development and disease. It exists in three methylation states: monomethylation (H3K27me1), dimethylation (H3K27me2), and trimethylation (H3K27me3), each with potentially distinct functions.	[130]
H4K20me3	Epigenetic modification that plays a role in gene regulation and chromatin structure. It is often associated with heterochromatin formation, which is a tightly packed form of DNA that is generally transcriptionally inactive. H4K20me3 is also involved in other cellular processes, including cell cycle regulation, DNA repair, and senescence.	[131]
H4K20me2	Epigenetic modification involved in DNA repair and DNA replication.	[132]
H4K20me	Epigenetic modification that plays a crucial role in various cellular processes, including DNA damage response, chromatin structure, gene expression, and cell cycle regulation. It exists in three forms: mono-, di-, and trimethylation (H4K20me1, H4K20me2, and H4K20me3). Each form has distinct functions and is regulated by different enzymes.	[133]
H2AK119ub	Histone modification primarily associated with gene repression. It is deposited on chromatin by the Polycomb Repressive Complex 1 (PRC1) and is involved in regulating various biological processes like development, tissue homeostasis, and potentially disease.	[134]
H3K14ac	Chromatin modification that plays a significant role in gene regulation and DNA repair. It is known to be associated with active gene expression and facilitates access to DNA by altering chromatin structure. Specifically, H3K14ac enhances the binding of chromatin remodeling complexes (RSC), which are crucial for DNA repair and transcription initiation.	[135]
H3K27ac	Epigenetic modification, primarily known for its role in activating gene expression and marking active enhancers. It is a key indicator of regions where DNA is more accessible and where transcription is likely to be occurring.	[136]
H3K9ac	Epigenetic modification that generally promotes gene transcription. It is associated with active promoters and enhancers, and helps to recruit proteins necessary for transcription.	[137]

## Data Availability

No new data were created or analyzed in this study. Data sharing is not applicable to this article.
