# Correction: Cevenini et al. Lytic or Latent Phase in Human Cytomegalovirus Infection: An Epigenetic Trigger. *Int. J. Mol. Sci.* 2025, *26*, 11554

**DOI:** 10.3390/ijms27135674

**Published:** 2026-06-24

**Authors:** Armando Cevenini, Pasqualino De Antonellis, Laura Letizia Mazzarelli, Laura Sarno, Pietro D’Alessandro, Massimiliano Pellicano, Serena Salomè, Francesco Raimondi, Maurizio Guida, Giuseppe Maria Maruotti, Marco Miceli

**Affiliations:** 1 Department of Molecular Medicine and Medical Biotechnology, University of Naples Federico II, 80131 Naples, Italy; pasqualino.deantonellis@unina.it; 2 CEINGE–Biotecnologie Avanzate Franco Salvatore, 80145 Naples, Italy; 3 Department of Public Health, University of Naples Federico II, 80131 Naples, Italy; lauramazzarelli@gmail.com (L.L.M.); pietro.dalessandro9676@gmail.com (P.D.); giuseppemaria.maruotti@unina.it (G.M.M.); 4 Department of Neuroscience, Reproductive Sciences and Dentistry, University of Naples Federico II, 80131 Naples, Italy; laura.sarno@unina.it (L.S.); pellican@unina.it (M.P.); mauguida@unina.it (M.G.); 5 Division of Neonatology, Department of Translational Medical Sciences, University of Naples Federico II, 80131 Naples, Italy; serena.salome@unina.it (S.S.); francesco.raimondi@unina.it (F.R.); 6 Regional Reference Center (CER) for Infectious Diseases in Obstetrics and Gynecology in Campania, 80131 Naples, Italy

In the original publication [1], the reference [146] (Sourvinos, G.; Morou, A.; Sanidas, I.; Codruta, I.; Ezell, S.A.; Doxaki, C.; Kampranis, S.C.; Kottakis, F.; Tsichlis, P.N. The downregulation of GFI1 by the EZH2-NDY1/KDM2B-JARID2 axis and by human cytomegalovirus (HCMV) associated factors allows the activation of the HCMV major IE promoter and the transition to productive infection. *PLoS Pathog.* **2014**, *10*, e1004136. https://doi.org/10.1371/journal.ppat.1004136.) was retracted in January 2026. This reference was cited only once and specifically in Section 8, Reversibility of the HCMV Genome from the Latent to the Lytic State, in Section 8.2, Latent Infection (Facultative Chromatin). The authors have replaced the retracted reference with an appropriate one without altering the scientific meaning of the sub-section. The new reference n. 146 is: Abraham, C.G.; Kulesza, C.A. Polycomb repressive complex 2 silences human cytomegalovirus transcription in quiescent infection models. *J. Virol.* **2013**, *87*, 13193–13205. https://doi.org/10.1128/JVI.02420-13.

The sentence containing the citation of the replaced reference (see above) was unclear. This sentence was in Section 8, Reversibility of the HCMV Genome from the Latent to the Lytic State, in Section 8.2, Latent Infection (Facultative Chromatin). The original sentence was: “Indeed, numerous studies have shown that their silencing results in a reduction in productive infection [145,146]” The authors replaced this sentence with: “Indeed, different studies have shown that PRC1 and PRC2 contribute to reducing productive infection [145,146]”.

The authors state that the scientific conclusions are unaffected. This correction was approved by the Academic Editor. The original publication has also been updated.
